# Supplementary material for: Effect of Temperature, Surface, and Medium Qualities on the Biofilm Formation of Listeria monocytogenes and Their Influencing Effects on the Antibacterial, Biofilm-Inhibitory, and Biofilm-Degrading Activities of Essential Oils
Source: Foods. 2025 Jun 14;14(12):2097. doi: 10.3390/foods14122097 (PMC12192137; doi:10.3390/foods14122097)
Supplement: Supplementary file 1 [file foods-14-02097-s001.zip › Supplementary Data S1A. Biofilm inhibition assay L6, MH-II, BHI + essential oils.pdf]

Results

ANOVA

ANOVA - Optical density (630nm)

Cases	Sum of Squares	df	Mean Square	F	p
Broth	0.005	1	0.005	1.182	0.277
Essential oils	2.434	57	0.043	9.694	< .001
Temperatures	0.130	2	0.065	14.774	< .001
Concentration (%)	0.077	1	0.077	17.447	< .001
Broth ✻ Essential oils	0.129	57	0.002	0.513	0.999
Broth ✻ Temperatures	0.032	2	0.016	3.637	0.027
Essential oils ✻ Temperatures	0.396	114	0.003	0.788	0.943
Broth ✻ Essential oils ✻ Temperatures	0.145	114	0.001	0.289	1.000
Broth ✻ Concentration (%)	0.008	1	0.008	1.906	0.168
Essential oils ✻ Concentration (%)	0.323	57	0.006	1.285	0.083
Temperatures ✻ Concentration (%)	0.038	2	0.019	4.295	0.014
Broth ✻ Essential oils ✻ Concentration (%)	0.082	57	0.001	0.327	1.000
Broth ✻ Temperatures ✻ Concentration (%)	0.032	2	0.016	3.646	0.027
Essential oils ✻ Temperatures ✻ Concentration (%)	0.270	114	0.002	0.538	1.000
Broth ✻ Essential oils ✻ Temperatures ✻ Concentration (%)	0.154	114	0.001	0.307	1.000
Residuals	3.065	696	0.004		

Note. Type III Sum of Squares
