# Supplementary material for: Effect of Temperature, Surface, and Medium Qualities on the Biofilm Formation of Listeria monocytogenes and Their Influencing Effects on the Antibacterial, Biofilm-Inhibitory, and Biofilm-Degrading Activities of Essential Oils
Source: Foods. 2025 Jun 14;14(12):2097. doi: 10.3390/foods14122097 (PMC12192137; doi:10.3390/foods14122097)
Supplement: Supplementary file 1 [file foods-14-02097-s001.zip › Supplementary Data S2A. Biofilm remove 1860 MH-II, BHI.pdf]

Results

ANOVA

ANOVA - Optical density (630nm)

Cases	Sum of Squares	df	Mean Square	F	p
Essential oil	3.538	58	0.061	26.242	< .001
Temperature (°C)	0.863	2	0.432	185.693	< .001
Concentration	0.019	1	0.019	8.296	0.004
Essential oil ✻ Temperature (°C)	1.108	116	0.010	4.109	< .001
Essential oil ✻ Concentration	0.529	58	0.009	3.924	< .001
Temperature (°C) ✻ Concentration	0.136	2	0.068	29.226	< .001
Essential oil ✻ Temperature (°C) ✻ Concentration	0.530	116	0.005	1.965	< .001
Broth	0.118	1	0.118	50.921	< .001
Essential oil ✻ Broth	0.392	58	0.007	2.908	< .001
Temperature (°C) ✻ Broth	2.475	2	1.237	532.281	< .001
Concentration ✻ Broth	0.146	1	0.146	62.861	< .001
Essential oil ✻ Temperature (°C) ✻ Broth	1.045	116	0.009	3.877	< .001
Essential oil ✻ Concentration ✻ Broth	0.414	58	0.007	3.070	< .001
Temperature (°C) ✻ Concentration ✻ Broth	0.404	2	0.202	86.839	< .001
Essential oil ✻ Temperature (°C) ✻ Concentration ✻ Broth	0.481	116	0.004	1.782	< .001
Residuals	3.291	1416	0.002		

Note. Type III Sum of Squares
