# Supplementary material for: Effect of Temperature, Surface, and Medium Qualities on the Biofilm Formation of Listeria monocytogenes and Their Influencing Effects on the Antibacterial, Biofilm-Inhibitory, and Biofilm-Degrading Activities of Essential Oils
Source: Foods. 2025 Jun 14;14(12):2097. doi: 10.3390/foods14122097 (PMC12192137; doi:10.3390/foods14122097)
Supplement: Supplementary file 1 [file foods-14-02097-s001.zip › Supplementary Data S3A. Biofilm remove in chicken shake.pdf]

Results

ANOVA

ANOVA - Optical density (630nm)

Cases	Sum of Squares	df	Mean Square	F	p
Hours	0.005	1	0.005	0.774	0.380
Temperature	0.065	1	0.065	9.480	0.002
Essential oil	0.402	57	0.007	1.032	0.424
Hours ✻ Temperature	0.145	1	0.145	21.169	< .001
Hours ✻ Essential oil	0.332	57	0.006	0.852	0.761
Temperature ✻ Essential oil	0.140	57	0.002	0.359	1.000
Hours ✻ Temperature ✻ Essential oil	0.286	57	0.005	0.733	0.918
Residuals	1.585	232	0.007		

Note. Type III Sum of Squares

Post Hoc Tests

Standard

Post Hoc Comparisons - Hours ✻ Temperature

		Mean Difference	SE	t	p_{tukey}
1 4	24 4	0.029	0.011	2.631	0.045
	1 23	0.012	0.011	1.076	0.704
	24 23	−0.030	0.011	−2.799	0.028
24 4	1 23	−0.017	0.011	−1.555	0.406
	24 23	−0.059	0.011	−5.430	< .001
1 23	24 23	−0.042	0.011	−3.875	< .001

Note. P-value adjusted for comparing a family of 4
Note. Results are averaged over the levels of: Essential oil
